# The Effectiveness of Personalized Nursing on Quality of Life in Cardiovascular Disease Patients: A Systematic Review and Meta-analysis

**DOI:** 10.1155/2023/4689732

**Published:** 2023-01-16

**Authors:** Min Yang, Na Ta, Xue Bai, Chengxi Wei, Chengshan Sun, Chunjuan Han

**Affiliations:** ^1^Nursing College, Tongliao City Hospital, Tongliao, Inner Mongolia, China; ^2^Inner Mongolia Key Laboratory of Mongolian Medicine Pharmacology for Cardio-Cerebral Vascular System, Tongliao, Inner Mongolia, China; ^3^Tongchuan People's Hospital Central Pharmacy, Tongchuan, Shaanxi, China

## Abstract

**Aims:**

This study aimed to examine the potential effectiveness of personalized nursing interventions on improving the heart-related quality of life of patients with CVDs versus an usual care.

**Design:**

A systematic review and meta-analysis. *Data Sources*. The study researched the article between January 2011 and December 2021 from four electronic databases: PubMed, Embase, Cochrane library, and Web of Science. *Review Methods*. Randomized controlled trials (RCTs) related to personalized nursing in CVDs population were included. The main variables were analyzed by standardized mean differences with 95% confidence intervals and heterogeneity was used by the *I*^2^ test and *P* value.

**Results:**

Of 734 studies, fourteen articles were eligible for this study. Personalized nursing significantly improved the quality of life [SMD = 0.39, 95% CI (0.29, 0.49)] with obvious heterogeneity (*P* = 0.000, *I*^2^ = 66.1%) which needs to be further subgroup analyzed. The nurse-led intervention was considered the main-related effect to influence the heterogeneity with value of 0.39 (I2 = 66.1%, *P* = 0.000; Group 1: I2 = 48.4%, *P* = 0.071, and Group 2: I2 = 0.0%,. In addition, related results of athletic ability and mental health and follow-up and education in the intervention had higher level of quality of life compared to the control group [SMD = 0.27, 95% CI (0.10, 0.44); SMD = 0.21, 95% CI (0.04, 0.37); SMD = 0.39, 95% CI (0.29, 0.49) and SMD = 0.28, 95% CI (0.11, 0.44)].

**Conclusion:**

Effectiveness studies of personalized care focus on more relevant outcomes have higher health outcomes, whereas evidence of the effectiveness of personalized nursing approach is still limited. Therefore, more and more high-quality RCT are needed.

## 1. Introduction

Cardiovascular diseases (CVDs) is the main reason for mortality with more than 17 million deaths each year (World Health Organization, 2019-40) and responsible for one-third of the deaths worldwide. In particular, 80% of CVD-related deaths were recorded and increasing in low- and middle-income countries [1]. In China, CVDs have become the most prevalent disease in aged over 65 years people with suffering health condition [2]. CVDs are a type of chronic illness which typically lead to a decline in physical capacities and emotional conditions, the patient becomes lonely and socially isolated which are the complex and long-time progressive nature of CVDs. They may cause high re-hospitalization and financial burden cost of medical care and eventually negatively influence a patient's health-related quality of life (HRQOL) [3].

Health-related quality of life covers multidimensional domains that are linked to the physical, emotional, psychological, and social functions which has the important role in the prevention of chronic disease relapse like cardiology sequel [4]. Quality of life has been identified as one of the main indicators of measurement of cardiovascular health outcome [5]. In general, cardiovascular diseases patients had low level of HRQOL, and patients with unhealthy lifestyle, being lack of confidence in treatment and prognosis and being failure to actively and strictly follow the doctor's advice to take medicine and rehabilitation training have been indicated to tend to be lower HRQOL [6].

For CVDs, some patients may ignore its continuous treatment throughout life and compliance behavior mainly by motor disturbances and emotional caring following their discharge from a healthcare institution, therefore, in order to improve the health status and treatment outcome related to quality of life, it is necessary to apply nursing theories integrated care interventions in patients after-treatment [7, 8]. The carers' roles in the supportive process are taken when the patients are ready to learn and do more something, but he/she cannot do it without help and guidance. Previous studies have been informed that skillful and personal nursing like provision of medical and psychological guidance to the patients is critical to improving their quality of life [9]. With continuous nursing, especially personalized care, as an extension of high-quality medial caring to the recovering, it mainly included providing education, counseling, emotional support, or help with accessing services by using information packages, limiting sodium intake, improve physical activity, fruit and vegetable consumption, and weight loss [9].

It should be noted that intense care by nurses for CVDs has contributed immensely in the complete success after hospital treatment [3]. As we know, treatment of CVDs play a key role in improving clinical outcomes, early physician follow-ups may reduce the risk of 30-day readmission for those with cardiovascular diseases [10]. However, due to the fact that heart failure is a chronic disease, the role of nurses had an meaningful effect on identification of problems and support of patients, dealing with the diagnosis, guiding their behavioral and lifestyle modifications for achieving more effective management, recurring, and even how to deal with death in the process of after-treatment [11, 12]. At present, the role of nurses has often been underestimated and left unnoticed through research in chronic after hospital treatment. Although a number of randomized controlled studies have confirmed that personalized care can reduce the negative emotion and promoting the recovery of physical function and in patients with post-CVDs and play a positive role in improving the quality of life. Few studies were to explore the details of nursing programs, contents, and results and its lack of reliable evidence-based measurements which limits the promotion of this program. Therefore, this study examined the potential effectiveness of personal care interventions in improving the HRQOL of patients with CVDs versus a usual care.

## 2. Materials and Analysis

### 2.1. Protocol and Registration

The study was reported following the guidelines of the Preferred Reporting Items for Systematic Reviews and Meta-Analyses (PRISMA 2020). We registered the study on PROSPERO under the registration number (CRD42022354872).

### 2.2. Search Strategy

The electronic network databases were researched in PubMed, EMBASE, Web of Science, and the Cochrane Library. The retrieval teams were mainly based on a combination of MeSH subject words and related-free words.

The search terms regarding the participant included (“Cardiovascular diseases” OR “Cardiovascular Disease” OR “Disease, Cardiovascular” OR “Diseases, Cardiovascular”). With “Nursing” OR “Nursings” OR “Caring” OR “Empathy” OR “Compassion” as the intervention searched terms. The type of study was limited “randomized controlled trial (RCT) OR placebo” in English. The measurement of outcome used for “Quality of life OR Health-Related Quality Of Life” in English and so on. Reference lists of studies and relevant systematic reviews were manually screened to identify further eligible research. The retrieval type or blinding were not limited, and the research time was conducted from January 1^st^, 2011 to December 31^st^, 2021. The detailed full electronic search strategy of Embase is shown in supplementary file. (Available here).

### 2.3. Inclusion and Exclusion Criteria

#### 2.3.1. Inclusion criteria

(1) Studies on patients with related cardiovascular and cerebrovascular diseases who were diagnosed by coronary heart disease, hypertension, heart failure and so on. There were no age or gender limits; (2) the intervention group was treated with personalized care, while the control group was treated with usual care; (3) the primary outcome was health-related quality of life as measured by reliable and validated instruments, with athletic ability and mental health as secondary outcomes (details of the indicators were shown in “Outcome indicators”).

#### 2.3.2. Exclusion Criteria

(1) Non-RCTs; (2) systematic reviews, meta-analysis, case reports, meeting abstracts, animals tests, and related commentaries; (3) inconsistent, incomplete, or ambiguous baseline data on disease and other associated characteristics of the participants; (4) Lack of original data, only partial abstracts or data provided, no full text, or no response to contacting the author.

#### 2.3.3. Intervention and Control Measurements

The intervention group was treated with personalized care, such as education, follow-up, rehabilitation exercise and so on. Meanwhile, the control group received only usual care

#### 2.3.4. Outcome Measurement Indicators

*(1) Primary Outcomes*. The retrieved studies contained primary and secondary indicators, with quality of life being the primary outcome measured in reliable and valid scales. The quality of life was measured at the time of baseline and the end of intervention, based on reliable and valid scales that have been used around the world including the following scoring tools: the 36-Item Short Form Health Survey (SF-36), SF-12, and WHOQOL-BREF.

SF-36 is a set of generic, coherent, and easily administered QOL measure. Its measures rely on patient self-reporting and are widely utilized for routine monitoring and assessment of care outcomes in adult patients. It comprises 36 questions which cover eight domains of health: physical activities, social activities, usual role activities, bodily pain, general mental health (psychological distress and well-being), usual role activities, vitality (energy and fatigue), and general health perceptions. Each scale is directly transformed into a 0–100 scale on the assumption that each question carries equal weight. The lower score means that the more disability.

WHOQOL-BREF, World Health Organization Quality of Life project 26-item instrument. The lower score means that the more disability.

*(2) Secondary Outcomes*. The secondary outcome measures with caring-related outcome included QOL-related outcomes like athletic ability, mental health, and the intervention methods like follow-up and education.

### 2.4. Data Extraction and Screening

After the selected studies were extracted and imported into Note Express for electronic and manual duplicate checks, [13] two researchers independently examined abstracts, screened, read, and excluded the irrelevant papers. Publications with inappropriate study type designs, incomplete results data, and full-text were removed. A full-text screening and data extraction were performed according to the above eligible inclusion and exaction criteria comprising participants, interventions, controls, outcomes, and study design framework (PICOS). Also, their differences will be resolved with the help of a third reviewer until a consistent conclusion and consensus were reached. The detailed information was extracted from the final eligible articles and recorded in a Microsoft Excel: Those sheets included details of the authors, year of publication, study design, characteristics of participants, intervention, control group, and outcomes (Table 1).

### 2.5. Data Analysis

Extracted data were analyzed by the Stata 16.0 software to perform meta-analysis. We used the chi-square test and the *I*^2^ statistic and *P* value to evaluate heterogeneity among the studies. If the outcome indicator is a continuous variable, we used the standardized mean difference (SMD) or mean difference (MD) and 95% confidence interval (CI) to analyze the studies. The MD and SMD were used as a summary statistic if all trails collected the same outcome indicator by the same scale. According to the results of quantitative analysis, we considered *P* < 0.05 and *I*^2^ < 50% to be statistically significant that meant good agreement and the fixed effect model was chosen.

To test the stability of the meta-analysis results of each index, we used the one-by-one elimination method to analyze the sensitivity of the main outcome indicators. To test the stability results of each index, a sensitivity analysis was conducted to investigate the potential source of heterogeneity and determine whether the final conclusion of the research is for a specific method or research design features used. We will further explore the effect of different detailed care approaches and related outcome indicators and other factors. They are analyzed based on the apparent homogeneity of results that can be qualitatively measured. Stata 16.0 software was used for sensitivity analysis, subgroup analysis, and sensitivity analysis and charts were drawn.

### 2.6. Publication Bias

Funnel chart is used to evaluate whether there is publication bias and Begg's test is used for the evaluation of potential publication bias.

## 3. Results

### 3.1. Selected Studies Quality Appraisal

**Figure 1:**
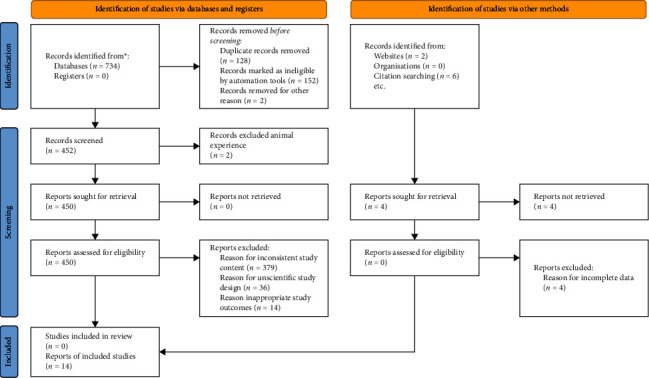
PRISMA 2020 flow diagram for new systematic reviews which included searches of databases, registers, and other sources.

Figure 1 presents the PRISMA 2020 flow diagram of this study. A total of 734 related articles that met the search criteria were collected from four English databases (PubMed: *n* = 24, Web of Science: *n* = 316, Embase: *n* = 154, Cochrane: *n* = 240). The retrieved articles were recorded in Note Express and 128 duplicated papers have been excluded by automatically screening. In addition, inappropriate studies like reviews, meta-analysis, and animal mechanism experiments (*n* = 156) were removed after reading the titles and abstracts. Furthermore, articles that did not meet the study inclusion standard, including 379 studies with inconsistent content, 36 studies with unreasonable design, and 21 inconsistent outcome indicators, they were eliminated after reading the full text. Finally, 14 documents were included in the quantitative meta-analysis.

### 3.2. Study Characteristics

The included studies had a total of 1562 patients (intervention group: *n* = 844 and control group: *n* = 718) covering the period from 2011 to 2021. The researched subjects were patients who were diagnosed with related cardiovascular disease, and there was no significant difference between groups for these outcomes. The age of patients varied from 18 to 85 years. The duration of intervention ranged from ten weeks to one year. Quality of life was categorized as a primary outcome measurement in all trials. The intervention group received the personalized care while the control group received the usual care or routine care. The included studies were conducted in the all of world, and all of the outcomes of quality of life were scored using reliable and valid ranking scales. Table 1 shows the characteristics of the included study.

### 3.3. Summary of the Quality and Bias Risk of the Trials

**Figure 2:**
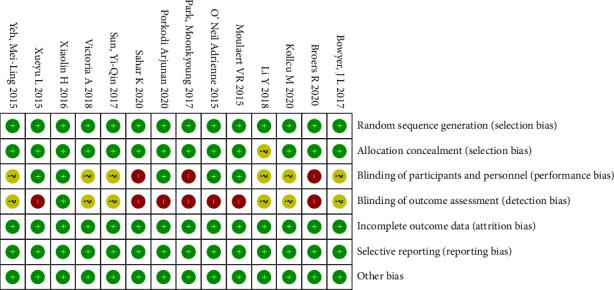
Risk of bias graph.

**Figure 3:**
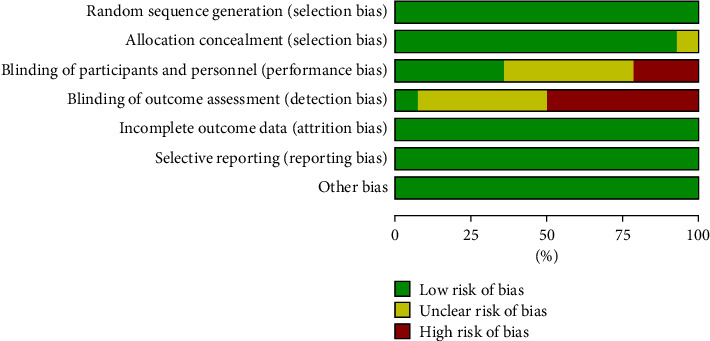
The summary for the risk of bias based on the selection criteria.

According to Cochrane collaboration risk of bias tools, most of the trails had relatively low risk of bias. All of the included studies had similar group characteristics at baseline. It was found that only 13 studies reported random sequence generation details, 5 included studies blind of their patients, investigators, or the assessors. The additional sources of bias in all trails were low due to inclusion criteria. Details of risk of bias are summarized in Figures 2 and 3.

### 3.4. Outcome Measures

#### 3.4.1. Effect of the Quality of Life

**Figure 4:**
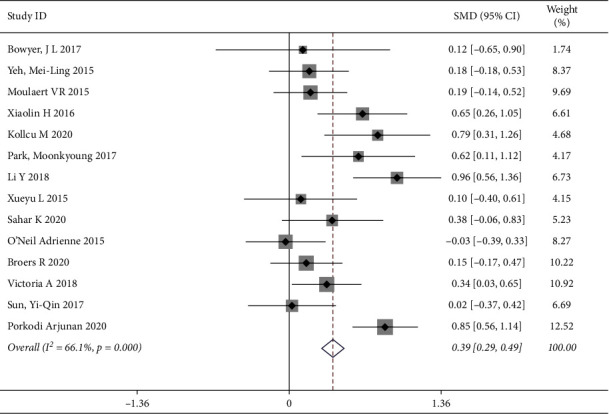
Forest map of scoring.

“Quality of life” was measured by standard and generic scales around the world, and was the primary outcome measure for all the included articles. We evaluated the differences in the effect of outcome between the intervention group (personalized care) and control care (usual care). 14 studies involving 1562 patients (844 patients in the intervention group and 718 patients in the control group) showed that, compared to the control group, the meta-analyses indicated that personalized care improved the level of quality of life [SMD = 0.39, 95% CI (0.29, 0.49)], and there was obvious heterogeneity (*P*=0.000, *I*^*2*^ = 66.1%); thus, the random effects model was used to analyze the data (Figure 4). The causes of the observed heterogeneity were further investigated by using the subgroup and sensitive analyses. To examine the origin of heterogeneity, we explored that detailed intervention can be used to distinguish subgroups of patients with cardiovascular and cerebrovascular diseases had different quality of life.

#### 3.4.2. Subgroup Analysis for Quality of Life

**Figure 5:**
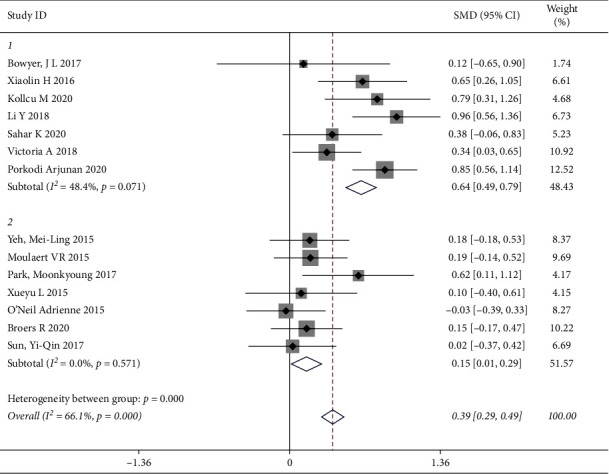
Forest map of quality of life for subgroups.

In this meta-analysis, the nurse-led intervention was considered as the main related effect to influence the heterogeneity, and the two subgroups (Group 1: being nurse-led intervention; Group 2: without nurse-led intervention) indicated that they were extremely heterogeneous. There was no heterogeneity within each subgroup as shown by an effective value of 0.39 (*I*^2^ = 66.1%, *P*=0.000; Group 1: *I*^2^ = 48.4%, *P*=0.071, and Group 2: *I*^2^ = 0.0%, *P*=0.571), suggesting that nurse-led intervention as a treatment may influence the heterogeneity and improve the QOL among cardiovascular disease patients. The results of the subgroup analysis could be carried out and found in Figure 5.

#### 3.4.3. Effect of Athletic Ability on Secondary Results in Cardiovascular and Cerebrovascular Patients

**Figure 6:**
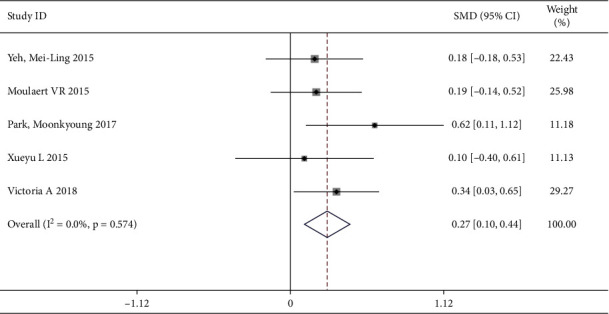
Forest map of the athletic ability.

A total of 5 trails [2, 3, 6, 8, 12] reported the athletic ability as the related result of QOL, involving 598 patients (the intervention group included 355 patients and the control group included 243 patients). There was no heterogeneity (*I*^2^ = 0.0%, *P*=0.574), thus, fixed effects model was applied for data analysis. The results indicated that the intervention group had higher quality of life than that in the control group by focusing on improving athletic ability, which showed a statistically significant difference [SWM = 0.27, 95% CI (0.10, 0.44)] (Figure 6).

#### 3.4.4. Effect of Mental Health on Secondary Results in Cardiovascular and Cerebrovascular Patients

**Figure 7:**
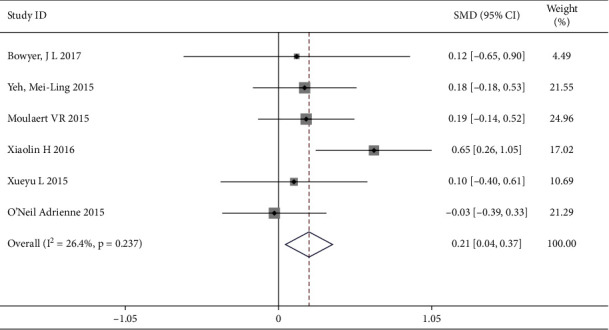
Forest map of the mental health.

A total of 6 trails [1–4, 8, 10] reported the mental health as the secondary result, involving 581 patients (the intervention group included 305 patients and the control group included 276 patients). There was no heterogeneity (*I*^2^ = 26.4%, *P*=0.237) and the fixed effects model was applied for data analysis. The results indicated that the intervention group had higher level of quality of life by increasing mental health, which showed a statistically significant difference [SWM = 0.21, 95% CI (0.04, 0.37)] (Figure 7).

#### 3.4.5. Effect of Follow-Up on Intervention in Cardiovascular and Cerebrovascular Patients

**Figure 8:**
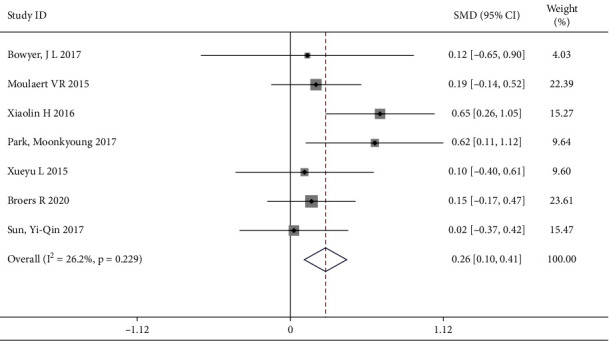
Forest map of the intervention of follow-up.

A total of 7 trails [1, 3, 4, 6, 8, 11, 13] reported the follow-up as the personalized care, involving 649 patients (the intervention group included 337 patients and the control group included 312 patients). There was no significant heterogeneity (I^2^ = 26.2%, *P*=0.229) and a fixed effects model was used. Meanwhile, a positive influence on improving quality of life was noted after the follow-up intervention [SMD = 0.39, 95% CI (0.29, 0.49)] (Figure 8).

#### 3.4.6. Effect of Education on Intervention in Cardiovascular and Cerebrovascular Patients

**Figure 9:**
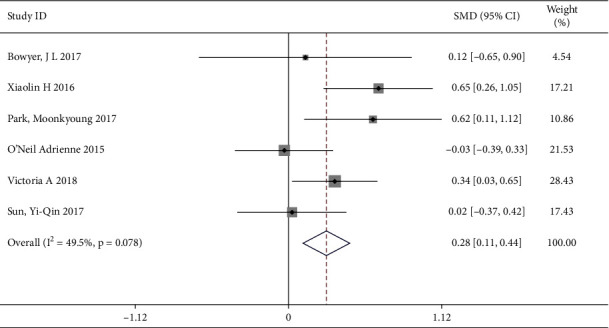
Forest map of the intervention of education.

A total of 6 trails [1, 4, 6, 10, 12, 13] reported the education as another personalized care, involving 623 patients (the intervention group included 366 patients and the control group included 257 patients). No significant heterogeneity was observed (*I*^2^ = 49.5%, *P*=0.078); therefore, a fixed effects model was used. The analysis showed a significant improvement effect on QOL in the intervention group compared to the control group by education care intervention [SMD = 0.28, 95% CI (0.11, 0.44)] (Figure 9).

### 3.5. Funnel Plot

**Figure 10:**
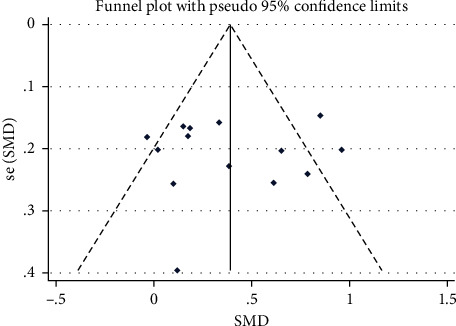
The funnel plot of quality of life by ranking.

The comparison-corrected funnel plot shows that most of the dots are symmetrically distributed on both sides of the *X* = 0 vertical line, suggesting that publication bias and small sample effects are less likely. (see Figure 10

## 4. Discussion

The occurrence of CVDs might bring physical or emotional disorders and further worsening or even being die. Caring or nursing offers in basic skills of physical activities and reduced negative emotional function which plays a positive role in the posthospital treatment and rehabilitation to improve the quality of life in CVDs patients [14]. The study aims to system review standardized and high-quality papers to explore the detailed approaches and outcomes of continuous personalized nursing intervention for improving the quality of life among CVDs patients. For secondary results, our results show that the intervention group has higher quality of life with focusing on athletic ability and mental health compared to the control group, in addition, the intervention group by application continuous education and follow-up care is higher in quality of life than in the control group with usual care which it has been confirmed in previous study.

Our main result showed that personalized caring had a significant improvement in quality of life compared to usual caring, which were consistent with previous studies [15]. The personalized caring is usually considered as an effective on the psychological adaptation and reduction of manipulative behaviors in patients with CVD [15]. Personalized nursing with nurse-patient communication could reduce disease burden, and negative emotions to rebuild their confidence may help improve clinical outcomes which may monitor and improve quality of life [15, 16]. In our subgroup analysis, we found that different detailed treatment approaches of nurse-led intervention influenced the heterogeneity of measurement QOL in our study. Previous studies showed that all the CVDs-related nursing intervention approaches and related outcomes had a greater or lesser impact on the QOL [14]. Nurse practitioner roles could assist patients improve social functioning, role functioning emotional, and mental health with achieving these health system goals for CVDs patients [17]. For the comparison between QOL and outcomes, it might be a matter of patients unintentionally believing that they had the best possible care when seeing a laded nurse instead of usual caring, regardless of clinical outcome, and the fact that the proportion of patient over time was higher in the usual care group compared with the nurse-led care group in previous studies. Currently some potential sources of bias and unreported outcomes may influence the heterogeneity and subgroup analysis for improving QOL is inconsistent and limited.

Our results also showed that the patients in the intervention group had low level of emotional and physical function. Nowadays, there is sufficient evidence to support the urgent implementation of nurse interventions aimed at encouraging physical activity for enhancing limb function and muscle strength to carry out the activities of daily life which could somehow influence the QOL (quality of life) [9]. In the whole process, the nurses will discuss to the patients with their profiles and assess their activity level according to the actual situation in order to raise awareness of self-monitoring, self-monitor, and self-efficacy for improving the patient's level of physical activity [19]. Regarding the emotion dimension, patients in the nursing stage are more aware of the condition and what will happen once relapsing after a period of treatment [20]. For these reasons, they may follow nurses' suggestions by enhancing confidence and optimistic emotion to improve prognosis [20]. As we know, emotional management was important for CVDs, with a vital role for the nurse specialist in terms of counseling and reassurance by focusing on patient activating patients to perform self-management, recovering from depression, may positively influence to change their actions more autonomously [15]. All the above research further supports the results of this current analysis.

According to the findings of our study, personalized nursing related the detailed approach of educational management and follow-ups can be effective in improving the quality of life of CVDs patients. For CVDs patients, the medical focus changes from “cure” to “care” which includes providing education support to families who usually provided detailed information and practical skills by using patient-directed goal setting and introducing the experience of role models that facilitated the participants' confidence in improving the health situation [21]. Obviously, this kind of care might be different from the “usual care.” For example, in one study, the effect of intervention on HRQoL was high assessed by long-timefollow-up [22]. For durable personalized caring, nurses could connect communication by spending long time to build trusting which are more suitable than other families or professionals as educators for chronic illness patients [23, 24].

## 5. Strength and Limitation

In this meta-analysis, we performed a detailed classification and analysis of the intervention group which will enhance reproducibility of the intervention. Meanwhile, this work included the inclusion of only RCTs, two authors assessed study quality ratings, adverse events were analyzed using the Cochrane and Grade, and subgroup analyses were performed to identify potential associations between intervention nursing methods and quality of life.

While this systematic review still has some limitations, firstly, due to many different specific established standardized intervention caring regimens and contain a small size of participants identified will increase the uncertainty of generalizability of these studies. In addition, most studies had follow-up care of no more than 1 year with being lacked the long-termfollow-up data, therefore, we did not perform the meta-analysis to assess the long-term effect of the improvement of quality of life for CVD patients. Finally, the QoL is a kind of subjective data, which are influenced by many factors such as the situation of understanding or communication and prone to be biased.

## 6. Conclusion

Nurse-led disease management program appears to be effective in improving the quality of life for patients with CVDs. Our study reviewed new and valuable insight from patients and nursers on the post-treatment of those CVDs primary care providers. This meta-analysis showed that patients with personalized caring who attended this program revealed higher levels of quality of life, physical activity, and emotional activity compared with the usual caring. However, the promising results should be cautiously interpreted and generalized. Large-scale, prospective, randomized controlled trials are still needed to verify the preliminary findings of the current study.

## Figures and Tables

**Table 1:** Summary of data abstracted from included studies.

NO	Author, year, country	Sample size (N; IG/CG)	Inclusion criteria	Intervention strategies	Control strategies	Length of intervention	Primary outcome (QOL)	Secondary outcomes
1	Bowyer, J L 2017 USA	22/9	Atrial fibrillation aged 52–73	nurse-led intervention	Usual care	6 months	SF-36 0–100	Medication adherence self-management process mental health

2	Yeh, mei-ling 2015 China	*N* = 123 IG *n* = 63 CG *n* = 60	Hypertension aged >20	Auricular massage	Usual care	10 weeks	SF-36 0–100	mental health

3	Moulaert VR 2015 Netherlands	*N* = 143 IG *n* = 76 CG *n* = 67	Cardiac arrest aged 48–72	Follow-up on nerves	Usual care	12 months	SF-36 0–100	mental health athletic ability

4	Xiaolin H 2016 China	*N* = 102 IG *n* = 51 CG *n* = 51	Cardiovascular disease	multidisciplinary supportive program	Usual care	3 months	SF-36 0–100	mental health

5	Kollcu M 2020 Turkey	*N* = 74 IG *n* = 37 CG *n* = 37	Hypertension aged 65–75	Nurse-led hypertension management	Usual care	20 weeks	SF-36 0–100	Medication adherence self-management process

6	Park, Moonkyoung 2017 South Korea	*N* = 64 IG *n* = 32 CG *n* = 32	Myocardial infarction Aged >45	Goal attainment theory-based educational programs	Usual care	24 weeks	SF-36	health-related behaviors

7	Li Y 2018 China	*N* = 110 IG *n* = 55 CG *n* = 55	Coronary artery aged >59	Comprehensive care	Usual care	3 months	SF-36 0–100	self-management process mental health

8	Xueyu L 2015 China	*N* = 61 IG *n* = 32 CG *n* = 29	Cardiovascular disease aged >75	Home-based exercise program	Usual care	3 months	SF-36 0–100	mental health athletic ability

9	Sahar K 2020 Iran	*N* = 64 IG *n* = 32 CG *n* = 32	Coronary heart disease aged >30	complex health intervention	Usual care	3 months	SF-12	Medication adherence self-management process

10	O'Neil Adrienne 2015 Australia	*N* = 123 IG *n* = 61 CG *n* = 62	Acute coronary syndrome aged >41	Telehealth intervention	Usual care	12 months	SF-12	mental health

11	Broers R 2020 USA	*N* = 150 IG *n* = 76 CG *n* = 74	Heart failure, coronary artery disease, hypertension aged >45	Advanced next generation ecosystem	Usual care	3 months	BREF	health-related behaviors

12	Victoria A 2018 Spain	*N* = 207 IG *n* = 152 CG *n* = 55	Hypertension aged >61	Physical activity (supervised group walking lessons, social and cultural activities)	Usual care	9 months	SF-36 0–100	Body pain

13	Sun, Yi-Qin 2017 China	*N* = 98 IG *n* = 48 CG *n* = 50	Cardiovascular disease aged >60	TCM health education intervention	Usual care	6 months	SF-36 0–100	self-management process

14	Porkodi Arjunan 2020 India	*N* = 200 IG *n* = 100 CG *n* = 100	Heart failure aged 31–88	Nurse-led CR Program	Usual care	3 months	SF-36 0–100	

Notes: Secondary outcomes: 1. Medication adherence; 2. self-management process; 3. mental health; 4. athletic ability; 5. health-related behaviors; 6. body pain.

## Data Availability

All the data supporting this meta-analysis are from previously reported studies and datasets, which have been cited. The processed data are available from the corresponding author upon request.

## Abbreviations

CVDs: Cardiovascular diseases
QOL: Quality of life
HRQOL: Health-related quality of life
OSF: Open science framework
SF-36: The 36-item short form health survey
WHOQOL-BREF: World Health Organization quality of life project 26-item instrument
PICOS: Participants, interventions, controls, outcomes, and study design framework
RCTs: Randomized controlled trials
non-RCTs: Nonrandomized controlled trials
SMD: Standardized mean difference
MD: Mean difference
CI: Confidence interval.

## Supplementary Materials

Supplementary Materials The detailed full electronic search strategy of Embase was shown in supplementary file. A full-text screening and data extraction were performed according to the principle of “Participants, Interventions, Controls, Outcomes and Study design” (PICOS). The 1^st^ and 2^nd^ step was to search “patients disease” with MeSH and its free words. The 3^rh^ step was to combine them both; The 4^th^ and 5^th^ was to search “intervention methods” with MeSH and its free words and 6^th^ was to combine the step of 4^th^ and 5^th^; The 7^th^ and 8^th^ used outcome of our research as search strategy with MeSH and its free words, and the 9^th^ was to combine them; The 10^th^ was to search articles with type of “Random Controlled Trails.” At last, we integrated all above search strategies at the 11^th^ step.
