# Supplementary material for: The Effectiveness of Personalized Nursing on Quality of Life in Cardiovascular Disease Patients: A Systematic Review and Meta-analysis
Source: Evid Based Complement Alternat Med. 2023 Jan 16;2023:4689732. doi: 10.1155/2023/4689732 (PMC9870693; doi:10.1155/2023/4689732)
Supplement: Supplementary Materials — The detailed full electronic search strategy of Embase was shown in supplementary file. A full-text screening and data extraction were performed according to the principle of “Participants, Interventions, Controls, Outcomes and Study design” (PICOS). The 1st and 2nd step was to search “patients disease” with MeSH and its free words. The 3rh step was to combine them both; The 4th and 5th was to search “intervention methods” with MeSH and its free words and 6th was to combine the step of 4th and 5th; The 7th and 8th used outcome of our research as search strategy with MeSH and its free words, and the 9th was to combine them; The 10th was to search articles with type of “Random Controlled Trails.” At last, we integrated all above search strategies at the 11th step. [file 4689732.f1.zip › Supplementary File.pdf]

Step	Search Strategy
#1	**'cardiovascular disease'**/exp
#2	**'cardiovascular disease'**:ab,ti OR **'disease, cardiovascular'**:ab,ti OR **'diseases, cardiovascular'**:ab,ti
#3	#1 OR #2
#4	**'empathy'**/exp
#5	**nursing'**:ab,ti OR **'nursings'**:ab,ti OR **'compassion'**:ab,ti OR **'caring'**:ab,ti
#6	#4 OR #5
#7	**'quality of life'**/exp
#8	**'life quality'**:ab,ti OR **'health-related quality of life'**:ab,ti OR **'health related quality of life'**:ab,ti OR **'hrqol'**:ab,ti
#9	#7 OR #8
#10	**'randomized controlled trial'**:ab,ti OR **'randomized'**:ab,ti OR **'placebo'**:ab,ti
#11	#9 AND (**2011**:py OR **2012**:py OR **2013**:py OR **2014**:py OR **2015**:py OR **2016**:py OR **2017**:py OR **2018**:py OR **2019**:py OR **2020**:py OR **2021**:py)

embase	#12	#3 AND #6 AND #9 AND #10
	#13	#12 AND (**2011**:py OR **2012**:py OR **2013**:py OR **2014**:py OR **2015**:py OR **2016**:py OR **2017**:py OR **2018**:py OR **2019**:py OR **2020**:py OR **2021**:py)
